# A Nationwide Survey Following the Devastating 2022 Floods in Pakistan: Current State of Knowledge, Attitude, and Perception Toward Climate Change and Its Health Consequences

**DOI:** 10.7759/cureus.63838

**Published:** 2024-07-04

**Authors:** Omar Irfan, Rubaid Azhar Dhillon, Mohammad Aadil Qamar, Salman Muhammad Soomar, Kainat Manzoor, Wajiha Rizwan, Hani Zarbaft Ali, Zara Arshad, Javaid Ahmed Khan

**Affiliations:** 1 Medicine, The Aga Khan University Hospital, Karachi, PAK; 2 Medicine, Riphah International University, Rawalpindi, PAK; 3 Clinical Research, ResearchX, Karachi, PAK; 4 Medicine, Ziauddin University, Karachi, PAK; 5 Community Health Sciences, The Aga Khan University Hospital, Karachi, PAK; 6 Medicine, Shaheed Mohtarma Benazir Bhutto Medical University, Larkana, PAK; 7 Pediatrics, University of Child Health Sciences, Lahore, PAK; 8 Medicine, Rehman Medical College, Peshawar, PAK; 9 Clinical Research, Shifa International Hospital, Islamabad, PAK

**Keywords:** climate change and its effect on life and health, climate change, flood, public and environmental health, public heath, natural disaster, pakistan

## Abstract

Background: Climate change (CC) persists as a critical public health concern, vividly demonstrated by Pakistan's severe unprecedented flooding from June to October 2022. The interplay between floods and CC highlights the urgent need to comprehend their complex dynamics. Given Pakistan's significant geographical vulnerability to CC events, assessing public awareness of CC becomes essential. This study aims to evaluate public knowledge, attitudes, and perception (KAP) regarding CC and its implications for overall health, reflecting onto governmental policies and community-based guidelines and enhancing preparedness for future natural calamities of similar magnitude.

Methods: A nationwide cross-sectional survey of Pakistani adults covering all provinces of the country was conducted from January to March 2023 using a prevalidated questionnaire. A purposive sampling strategy was used to enroll participants in the study. Where appropriate, the chi-square test or Fisher’s exact test was used to compare KAP among the sociodemographic groups. Multivariate analysis was used to explore predictors of knowledge. Crude and adjusted odds ratios (ORs) were calculated considering a p value of ≤0.05 as significant.

Results: Among the 714 respondents, 265 (37.1%) of the respondents’ residential areas were affected by the floods in Pakistan. A total of 663 (92.9%) of the participants had heard of CC, with 302 (42.3%) choosing "social media/WhatsApp" as their source of information. Increased flooding and changes in rainfall patterns were selected by 679 (95.1%) and 661 (92.6%) participants, respectively, as the most recognized CC. "Deforestation" was the most indicated reason for CC by 675 (94.5%) participants. Multivariate analysis revealed that females (OR: 1.31, 95% CI: 1.16-2.00; p < 0.001), individuals who were affected by recent floods (OR: 1.13, 95% CI: 1.05-3.34; p = 0.003), and individuals who were medical students/healthcare workers (OR: 1.49, 95% CI: 1.24-2.48; p < 0.001) had greater knowledge of CC than their counterparts.

Conclusions: The study reported an encouraging prevalence of knowledge of CC, positive attitudes, and practices toward CC, with an interest in learning and doing more to address the health effects of CC. With the ongoing global CC and a monsoon season forecast of similar intensity for the years to come in Pakistan, identifying groups with less knowledge of CC warrants a targeted education program to maximize awareness. Based on the study findings, social media platforms and interventions in educational institutes should be essential to mitigate the CC events in Pakistan and other vulnerable regions in the area.

## Introduction

An estimated 971 million people worldwide live in regions highly susceptible to climate hazards, with a considerable portion of these vulnerable populations clustered in less developed nations or low- and middle-income countries [1]. This population further consists of the most vulnerable groups, which are dependent on agricultural or coastal livelihoods affected by climate change (CC) [2]. The environmental consequences of CC, such as rising temperatures, droughts, sea-level rise, flooding, and forest fires, have continued to affect human health [3]. Several larger studies and reviews have shown negative health effects of CC, including an increase in infections, respiratory disorders, heat stress-related morbidity, deaths, food insecurity, and adverse health impacts caused by sociopolitical instability and conflicts that arise after CC devastation events such as floods [3-5].

Pakistan ranks among the five most CC-affected countries globally, attributed to its documented history of severe weather events between 1999 and 2018 [6]. The devastating impacts of CC and extreme weather can exacerbate the existing problems of poverty and food insecurity in the country. From 1998 to 2018, Pakistan suffered approximately 152 extreme weather events, with approximately 10,000 casualties, and suffered $3.8 billion in economic losses [7]. Despite the literature documenting the impact of CC in Pakistan, including increased temperatures, unpredictable rainfall, rapid glacial melt, and reduced crop productivity, there remains limited understanding of how Pakistanis perceive CC and its extremes, how CC affects their lives and livelihoods, and the actions they are currently or willing to take to adapt [8,9].

The country has experienced many floods, with the most severe flooding from June to October 2022, which has been described as one of the worst floods in the country's history. It has raised significant concerns about the existing infrastructure and management protocols for overcoming such disasters. One year after the 2022 monsoon season began, floods affected at least 33 million people, displaced at least 7.9 million people, and caused at least 1,718 casualties [10]. According to reports, floods affect nearly 15% of Pakistan’s rice and 40% of its cotton crops, and more than 1.2 million livestock are killed, potentially leading to severe food shortages. It is estimated that the 2022 flooding led to $14.9 billion in damage and $15.2 billion in economic losses [11].

Among the many public health concerns raised after the 2022 floods, including hepatitis, dehydration, and skin problems, a notable surge of 140,000 malaria cases occurred in the Sindh Province of Pakistan during the first three weeks of September 2022 [12].

Considering the recent 2022 and 2023 floods across Pakistan and the absence of comprehensive assessments on CC knowledge within the country, the findings of this study hold promise in informing policymakers and governmental entities about the local population's understanding of CC and strategies to mitigate its impacts. This study sought to gather data on the knowledge, attitudes, and perceptions (KAP) regarding CC and climate-sensitive diseases among the general population of Pakistan, including those residing in areas severely affected by the 2022 floods.

## Materials and methods

Study population and timeline of data collection

A cross-sectional survey was conducted among the residents of Pakistan from January 1, 2023, to March 1, 2023, to assess their KAP regarding CC and its effects on health. The targeted population included adult Pakistan residents who were at least 18 years of age. An information sheet was provided to all participants before they signed the consent to take part in the survey. The protocol of the study was approved by the Ethical Review Committee (ERC) of Islamic International Medical College, Rawalpindi, Pakistan (ref no. Riphah/IIMC/ERC/560), and an acknowledgment of consent was appended to the survey for every participant. This study was conducted in accordance with the principles of the Declaration of Helsinki.

Sampling strategy and sample size of the enrolled participants

A purposive sampling strategy was used to enroll participants in the study. We determined the sample size for this research by estimating the prevalence of individuals' awareness of CC. We assumed that approximately 50% of the population possessed some level of knowledge about CC (p = 0.5) [13]. The sample size was calculated considering a 95% confidence interval and 80% power, and the sample size calculated was 683. When accounting for a 10% nonresponse rate, the final sample size was 714.

The formula and calculation are as follows:

n = [{Z^2^a/2(pq)}/E^2^]*design effect

The values for the calculation were as follows:

Z^2^(a/2) = 1.96 at 95% CI

p = proportion of those who have CC knowledge = 0.50

q = proportion of those who do not have CC knowledge = 0.50

E^2 ^= precision (0.04)^2^

Design effect = 2

The questionnaire administered and the method of data collection

A self-administered questionnaire, designed after passing through previously validated questionnaires, was formulated as a tool for data collection [13,14]. Both online and in-person media were adopted to collect responses. The online dissemination of the questionnaire took place via social media platforms such as Google Forms, where distribution among various groups was conducted. The questionnaire was initially developed in the English language but was also translated into Urdu by the authors (OI and MAQ) to interview participants who were not fluent in English, where the translation was validated by the senior authors (WR and JAK) and cross-checked via Google Translate. The survey comprised 23 questions and was divided into sections on sociodemographic characteristics, knowledge of CC, and attitudes toward and perceptions of CC. Sections of the questionnaire included Likert scale responses (3 points); some used the multiple-response approach per question.

Validity and reliability of the questionnaire used for data collection

A pilot study of 20 individuals was conducted to assess compliance and clarity and to ensure relevance, following which necessary changes were made accordingly. These responses were omitted from the final analysis of this manuscript. Cronbach’s alpha and inter- and corrected-item total correlations were computed to assess the internal consistency. To evaluate the convergent validity of the tool, we calculated the Pearson correlation coefficient. The Cronbach’s alpha of the tool was 0.86 and did not increase when any item was removed from the analysis. All items in the tool were within the acceptable range, and none of the items exhibited a weak correlation for corrected item-total correlations, i.e., <0.3. No negative correlation was detected among the demographics and other covariates in the tool based on the Pearson correlation coefficient, i.e., p < 0.001.

Statistical analysis of the data collected

The collected data were transferred to the analytical software StataCorp. 2023. Stata Statistical Software: Release 17 (StataCorp LLC, College Station, TX), after a series of logical and range checks and data wrangling. The median and interquartile ranges were computed for age. The normality of the variables was assessed using the Shapiro-Wilk test. Sociodemographic variables and participant responses are summarized and depicted in frequency tables. The associations between sociodemographic variables and CC KAP, as well as practices, were explored through logistic regression. Multivariate binary logistic regression models were subsequently applied to delve deeper into the significant predictors of knowledge, attitudes, and practices. Crude and adjusted odds ratios were calculated considering a p value of ≤0.05 as significant. The analysis was performed, and graphs were obtained using StataCorp. 2023. Stata Statistical Software: Release 17.

## Results

Out of a total 900 questionnaires administered, 714 questionnaires were completed reflecting a survey completion rate of 79.3%.

Baseline characteristics of participants

Of the 714 participants, the median age was 25 years, ranging from 18 to 70 years. Participants were recruited from all provinces of Pakistan, with 301 (42.2%) from Sindh, 195 (27.3%) from Punjab, 87 (12.2%) from Khyber Pakhtunkhwa, and 77 (10.8%) from Balochistan (Table 1). A total of 413 (57.8%) females participated, while 265 (37.1%) respondents answered "Yes" to the question, "Was your area of living affected by recent floods in Pakistan?" A total of 432 (60.5%) respondents were bachelor/university graduates, and 259 (36.3%) participants were currently enrolled as students when asked about their employment status. Approximately 490 (68.7%) participants were either students or professionals in the healthcare field. The demographic and socioeconomic characteristics of the participants are described in Table 1.

**Table 1:** Sociodemographic characteristics of the participants (n = 714) Age: median = 25 years old; range = 18-70 years old KPK: Khyber Pakhtunkhwa

Sociodemographic data		Frequency, n (%)
Gender	Male	301 (42.2)
	Female	413 (57.8)
Location/province	Sindh	302 (42.3)
	Punjab	195 (27.3)
	KPK	87 (12.2)
	Balochistan	77 (10.8)
	Islamabad	53 (7.4)
Education	Master or Doctor of Philosophy	154 (21.6)
	Bachelor/university graduate	432 (60.5)
	Diploma	3 (0.4)
	Inter/A level	109 (15.3)
	Primary/secondary schooling	13 (1.8)
	Uneducated	3 (0.4)
Occupation	Businessman/woman (self-employed)	26 3.6)
	Government servant	116 (16.3)
	Private sector employee	156 (21.9)
	Retired	7 (1.0)
	Student	259 (36.3)
	Unemployed	147 (20.6)
	Unskilled worker	3 (0.4)
Student or professional in medicine/healthcare	Yes	490 (68.6)
	No	224 (31.4)
Monthly income (Pakistani rupees)	Monthly income (Pakistani rupees)	30 (4.2)
	<3,000	41 (5.7)
	3,000-15,000	38 (5.3)
	16,000-30,000	80 (11.2)
	31,000-60,000	203 (28.4)
	>60,000	322 (45.1)
Was your area of living affected by the recent floods in Pakistan?	Yes	265 (37.1)

Knowledge of CC

Of the 714 respondents, 663 (92.9%) expressed knowledge of CC, with "social media/WhatsApp" being the most common source of information for 302 (42.3%) participants (Table 2). When the participants were asked about the types of CCs, "increased flooding" was indicated by 679 (95.1%) participants, with "change in rainfall pattern" as the second most common type of CC indicated by 661 (92.6%) participants. Table 2 highlights the causes/responses for CC, where "Deforestation" was the most indicated cause by 675 (94.5%) participants.

**Table 2:** Participants' knowledge about CC (n = 714) *n = 714 percentage total is more than 100% due to multiple responses CC: climate change

Characteristic		Frequency, n (%)
Have you heard of what CC means from any source?	Yes	663 (92.9)
The main source of your information on CC	Social media/WhatsApp	302 (42.3)
	Television/radio	110 (15.4)
	Teachers/education	84 (11.8)
	Websites/online courses	42 (5.9)
	Research articles and conferences	34 (4.8)
	Newspaper	31 (4.3)
	Family members/friends/neighbors	21 (2.9)
	Personal involvement in training	10 (1.4)
	Nongovernmental organization workers	3 (0.4)
	I do not know	77 (10.8)
Type of CC*	Increased flooding	679 (95.1)
	Change in rainfall pattern	661 (92.6)
	Increased heatwaves	647 (90.6)
	Increased cold waves	384 (53.8)
	Increased number of cyclones	304 (42.6)
	I do not understand/I do not know	129 (18.1)
Causes or reasons for CC*	Deforestation	675 (94.5)
	Emissions from industrial activities	649 (90.9)
	Transport	647 (90.6)
	Burning fossil fuel	629 (88.1)
	Waste generation	623 (87.3)
	Overconsumption of natural resource	622 (87.1)
	Population growth	589 (82.5)
	Building sector	525 (73.5)
	Industrial agriculture	493 (69.1)
	God's will	449 (62.9)
	Increase in livestock farming	319 (44.7)

**Figure 1:**
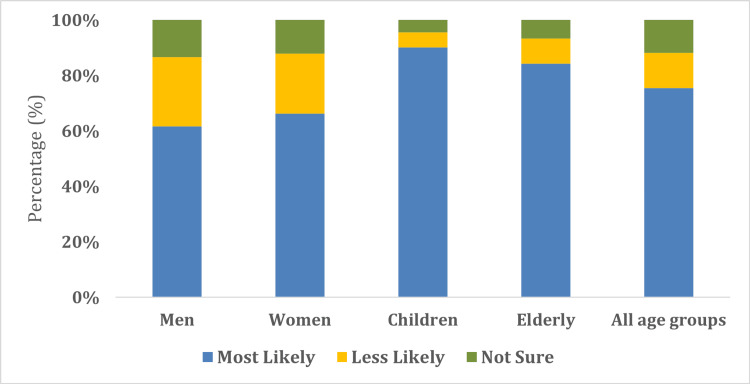
Human groups most affected by CC CC: climate change

**Figure 2:**
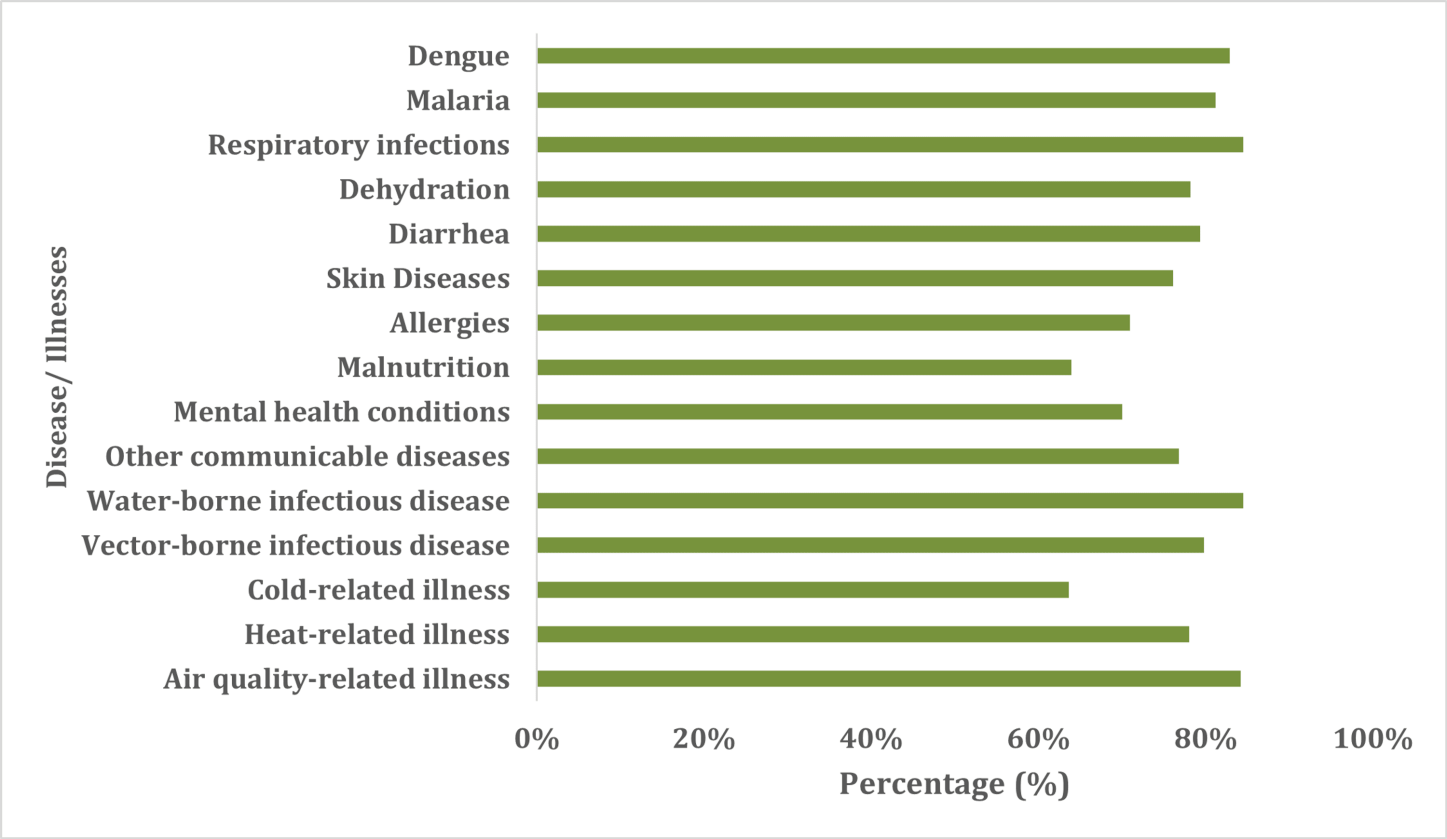
Impact of CC on human health and illnesses CC: climate change

Table 3 highlights the participants' knowledge and awareness of the impact that CC has on the environment and human health. Of the 714 participants, 637 (89.2%) of the population thought that they had experienced a "change in rainfall in the last 10 years because of CC." Furthermore, 582 (81.5%) participants indicated that they had experienced "increased episodes of floods and cyclones in the last 10 years" due to CC (Table 3). Figure 1 highlights that approximately 90.0% of the study participants thought that "children" were the "most affected by CC" among all age groups. Finally, most of the participants indicated that "respiratory infections" and "typhoid" had the most significant "impact on human health and illness due to CC" (Figure 2).

**Table 3:** Participants' knowledge and awareness of the impact of CC on the environment and human health (n = 714) CC: climate change

Characteristic	Frequency, n (%)
Change in rainfall pattern in the last 10 years	637 (89.2)
Increased episodes of floods and cyclones in the last 10 years	58 (81.5)
Increase in cases of new types of diseases	579 (81.1)
Death from disease-carrying vectors: malaria, dengue, chikungunya, etc.	558 (78.2)
Increased healthcare expenditure after an extreme weather event	556 (77.9)
Increased episodes of forest fires in the last 10 years	552 (77.3)
Increased health risk due to increase in salinity	532 (74.5)
Change in seawater level in the last 10 years	529 (74.1)
Reduced food crop production in the last 10 years	528 (74.0)
Increase in cases of mental illness: depression, anxiety, suicides, etc.	527 (73.8)
Increase climate-induced migration	522 (73.1)
Increase in cases of allergies	521 (73.0)
Scarcity of freshwater due to an increase in salinity	509 (71.3)
Increased episodes of drought in the last 10 years	476 (66.7)
Increase in social conflicts	469 (65.7)
Increased salinity of freshwater and/or groundwater in the last 10 years	468 (65.6)
Death from drowning in the last 10 years	362 (50.7)
Death from snake bite in the last 10 years	239 (33.5)

Attitude toward CC

Regarding the attitude of the study participants toward the impact of CC, almost 70.0% (n = 494) of the participants selected "high interest" for the questions "to know more about the impacts of CC on health impacts" and "to know more about the impacts of CC on health infrastructure." The greatest interest (76.9%) was noted in the question "to support green and healthy hospital design and construction" (Table 4).

**Table 4:** Participants' attitudes toward the impact of CC (n = 714) CC: climate change

Characteristic		Frequency, n (%)
To know more about the impacts of CC on health impacts	Low interest	30 (4.2)
	Medium interest	190 (26.6)
	High interest	494 (69.2)
To know more about the impacts of CC on health infrastructure	Low interest	42 (5.9)
	Medium interest	202 (28.3)
	High interest	470 (65.8)
To know more about the role of the government	Low interest	67 (9.4)
	Medium interest	207 (28.9)
	High interest	440 (61.6)
To know more about the role of nongovernmental institutions	Low interest	76 (10.6)
	Medium interest	248 (34.7)
	High interest	390 (54.6)
To know more about the linkages between infectious disease outbreaks and CC (e.g., SARS-CoV-2)	Low interest	36 (5.0)
	Medium interest	154 (21.6)
	High interest	524 (73.4)
To explore the role of health professionals in creating awareness of CC among the public	Low interest	34 (4.8)
	Medium interest	172 (24.1)
	High interest	508 (71.1)
To know more about building health systems that can withstand extreme climatic events and also reduce the carbon footprint	Low interest	42 (5.9)
	Medium interest	177 (24.8)
	High interest	495 (69.3)
To prioritize environmental health at your workplace/in your practice	Low interest	30 (4.2)
	Medium interest	141 (19.8)
	High interest	543 (76.0)
To substitute harmful chemicals with safer alternatives	Low interest	34 (4.8)
	Medium interest	170 (23.8)
	High interest	510 (71.4)
To implement energy efficiency and clean, renewable energy generation	Low interest	34 (4.8)
	Medium interest	172 (24.1)
	High interest	508 (71.1)
To purchase and serve sustainably grown, healthy food	Low interest	36 (5.0)
	Medium interest	166 (23.3)
	High interest	512 (71.7)
To support green and healthy hospital design and construction	Low interest	22 (3.1)
	Medium interest	143 (20.0)
	High interest	549 (76.9)
To buy safer and more sustainable products and materials	Low interest	27 (3.8)
	Medium interest	139 (19.5)
	High interest	548 (76.7)
To know more about the role of health professionals and workers	Low interest	35 (4.9)
	Medium interest	149 (20.9)
	High interest	530 (74.2)

Perception and practice related to CC

In addition, participants were asked about their practices related to CC and its impact. Table 5 shows that 524 (73.4%) participants admitted having discussed CC and its health effects with their colleagues. The participants indicated the least interest in researching air pollution and its impacts on health (Table 5). A total of 163 (22.8%) participants reported that "COVID-19 was caused by CC." COVID-19 recovery plans were favored to "be based more on activities that give priority to the health of the citizens" by 622 (87.1%) participants (Table 6). Finally, 663 (92.9%) participants would like to see "Better patient care facilities" as improvements in the health infrastructure to cope with future CC-related problems (Tables 5, 6).

**Table 5:** Participants' perceptions related to CC and its impact (n = 714) CC: climate change

Characteristic	Frequency, n (%)
Discussed about CC and its health effects with my colleagues	524 (73.4)
Educated the public about CC and its potential impacts on health	450 (63.0)
Encouraged or helped people to use active transportation such as walking and cycling	447 (62.6)
Educated the healthcare facility to adopt renewable energy sources	355 (49.7)
Involved in the CC-related awareness campaigns	311 (43.6)
Conducted research on CC and its impacts on health	257 (36.0)
Involved in air pollution-related awareness campaigns	251 (35.2)
Conducted research on air pollution and its impacts on health	244 (34.2)

**Table 6:** COVID-19 and future implications for CC (n = 714) CC: climate change; PPE: personal protective equipment

Characteristic		Frequency, n (%)
Do you think COVID-19 was caused because of CC?	Yes	163 (22.8)
Do you think the health infrastructure is prepared to deal with a mass epidemic in the future?	Yes	214 (30.0)
Do you think there were adequate preparations to deal with COVID-19?	Yes	207 (29.0)
What improvements in the health infrastructure would you like to see to cope with future CC-related problems?	Better patient care facilities	663 (92.9)
	Better emergency care	649 (90.9)
	Better building structure	591 (82.8)
	A regular supply of water	648 (90.8)
	Regular supply of electricity	621 (87.0)
	A regular supply of medicines and PPE kits	650 (91.0)
	Advanced information about the diseases	651 (91.2)
	Better coordination among departments	645 (90.3)
Do you think post-COVID-19 recovery plans should be based on activities that give priority to the health of the citizens?	Yes	622 (87.1)
Do you think post-COVID-19 recovery plans should be based on activities that give priority to the economy of the nation?	Yes	485 (67.9)
Do you think post-COVID-19 recovery plans should focus on conserving and protecting the environment in a strict manner?	Yes	574 (80.4)

Association of knowledge of CC with demographic variables

Multivariate binary logistic regression revealed that female sex, being a medical student/healthcare worker, and being in a residential area affected by the flood were significant factors for a higher knowledge of CC, as shown in Table 7. The odds of knowing about CC were 1.31 times higher for females than for males (95% CI: 1.16-2.00; p < 0.001). The odds of knowing about CC were 1.49 times greater (95% CI: 1.24-2.48) for medical students or healthcare workers than for non-healthcare professionals (p < 0.001). Finally, the odds of knowing about CC were 1.13 times greater (95% CI: 1.05-3.34) when the area was affected by recent floods (p = 0.0029) (Table 7).

**Table 7:** Multivariable analysis cORs and aORs with 95% CIs *Significant at the univariate level **Significant at the multivariate level cORs: crude odds ratios; aORs: adjusted odds ratios; CI: confidence interval; KPK: Khyber Pakhtunkhwa

Characteristics		cOR (95% CI)	P value	aOR (95% CI)	P value
Gender	Male	1	0.0025*	1	<0.001**
	Female	1.29 (1.08-1.96)		1.31 (1.16-2.00)	
Age (years)	18-27	1	0.623	-	-
	28-37	0.92 (0.55-1.53)		-	
	38-47	1.61 (0.47-5.43)		-	
	48-57	1.55 (0.45-5.25)		-	
	>58	0. 46 (0.11-1.77)		-	
Education	Masters/PhD	1	0.866	-	-
	Bachelor/university graduate	1.48 (0.98-3.20)		-	
	Diploma holder	0.87 (0.67-2.63)		-	
	Inter/A level	0.95 (0.74-2.70)		-	
	Primary/secondary schooling	0.62 (0.55-2.49)		-	
	Uneducated	0.55 (0.38-2.32)		-	
Occupation	Student	1	0.711	-	-
	Businessman/woman (self-employed)	0.91 (0.48-4.01)		-	
	Government servant	1.05 (0.77-4.63)		-	
	Private sector employee	1.23 (0.88-4.70)		-	
	Retired	1.13 (0.95-4.49)		-	
	Unemployed	0.86 (0.58-4.20)		-	
	Unskilled worker	0.93 (0.78-2.02)		-	
Location/province	Sindh	1	0.378	-	-
	Punjab	2.86 (1.07-4.44)		-	
	KPK	2.23 (0.72-3.90)		-	
	Balochistan	1.80 (0.68-3.54)		-	
	Islamabad	3.33 (1.39-5.60)		-	
Student or professional in medicine/healthcare	No	1	0.0001*	1	<0.001**
	Yes	1.68 (1.34-3.48)		1.49 (1.24-2.48)	
Was your area of living affected by the recent floods in Pakistan?	No	1	0.0001*	1	0.0029**
	Yes	1.14 (1.03-2.98)		1.13 (1.05-3.34)	

## Discussion

This study unveils the prevalence of various aspects of KAP related to CC and its health effects among the general population of Pakistan. Overall, a significant proportion of respondents demonstrated adequate knowledge, positive attitudes, and adaptive perceptions concerning CC, expressing a keen interest in furthering their understanding and efforts to mitigate its impacts. Pakistan finds itself particularly vulnerable in the global picture of worsening CC events. It is primarily due to the region's limited resources, weak policy structures, and socio-political challenges. Consequently, Pakistan continues to face challenges in the forms of loss of agricultural land, increased temperatures, and widespread, unpredictable floods [15].

In recent years, the significant glacier melting in Pakistan's northern regions and abnormal rainfall during the 2022-2023 monsoon season triggered catastrophic floods [15]. Given Pakistan’s vulnerability to CC, particularly as an agrarian economy, local knowledge and awareness of CC and its impact on health are crucial. The study revealed an encouraging prevalence of CC awareness, with 663 (92.9%) participants reporting awareness regarding CC.

Interpreting the results of knowledge and awareness

The devastating floods experienced in Pakistan in 2022 triggered a surge in public awareness regarding the human impact on CC. Our study reflected an overall higher level of CC knowledge, with 490 (68.6%) participants being healthcare professionals or students. Healthcare workers who directly witness the repercussions of CC on patients possess a heightened awareness of CC, which agrees with previous analyses from the United States of America, where healthcare professionals exhibited greater knowledge than nonhealthcare participants [16]. This study revealed a greater level of knowledge about CC in Pakistan than in other developing countries, such as Bangladesh (54%), Nigeria (54%), and Nepal (51.3%) [13,17,18]. However, the main source of information about CC in these countries was consistent with our findings. A similar study on CC was conducted in Pakistan with a smaller sample size, where the level of knowledge was reported to be 55% among the participants [19].

In this study, more than 90% of the participants believed that deforestation and transportation are the main causes of CC today. These findings overlap with those of other CC studies conducted in Pakistan and India, where deforestation and transportation were known to be the greatest contributors to CC by nearly 90% of the research participants [19,20].

In this study, younger participants expressed greater concern about CC than older individuals. A study conducted in the Czech Republic reported that 80.0% of participants, specifically adolescents, had more knowledge than adult participants [21].

Interpreting the impact of CC on health

Changing climatic conditions have contributed to the emergence of various infectious diseases, with 163 (22.8%) participants in this study attributing COVID-19 to CC. Widespread outbreaks of Zika and chikungunya viruses in the Americas are examples, with uncertain prospects for future expansion [22]. Modeling studies suggest that as global warming persists, current CC trends favor the spread of vector-borne diseases, potentially expanding vectors into new geographical areas [22].

Measures that need to be taken to address CC and its health impacts

Most participants in this study described interest in taking measures to address CC and its health impacts. While 524 (73.4%) participants initiated discussions about CC and its health effects with family and friends, only 36% researched this topic. Notably, among those directly affected by floods in Pakistan, only 257 (36.0%) participants showed interest in learning more about CC. This finding aligns with studies indicating that individuals who have experienced the effects of CC firsthand tend to exhibit greater concern and engage in adaptive behaviors [23,24]. Additionally, research suggests that the magnitude and severity of weather events influence the level of response to CC concerns [25].

To prepare for future heavy rainfall, such as those expected to occur in 2024 and beyond, the government needs to implement comprehensive water containment plans. A study conducted by the International Centre for Diarrheal Diseases Research in Bangladesh compared mortality rates between individuals residing inside and outside a flood-control embankment. The study revealed a 29% higher mortality rate in communities without flood protection [26].

In low-lying areas of Pakistan, communities should be educated about CC, and flood control structures must be constructed to safeguard these communities. Additionally, access to healthcare should be prioritized, which calls for the construction of additional medical centers to reduce mortality rates during such incidents. The most recent cyclone, Cyclone Biparjoy 2023, was a direct result of rising sea surface temperature, which posed a threat to several communities along the shores of Karachi, Pakistan [27]. Similarly, a study led by Indian researchers reported an increase in the frequency of cyclones in the Arabian Sea over the past four decades due to CC [28]. According to their findings, the government needs to take appropriate actions to protect these communities in the event of a disaster.

The literature also notes dengue being most prevalent in areas with warm temperatures and heavy rainfall, creating an ideal environment for mosquitoes to thrive and for viruses to replicate [29]. Therefore, the floods in Pakistan, alongside numerous stagnant pools of floodwater from poor infrastructure, provide fertile ground for the spread of dengue among the population. Furthermore, unregulated sewage disposal systems can contaminate clean water sources, contributing to the high incidence of diarrhea in flood-affected areas.

The health implications of CC are vast and encompass a range of factors. These include heat waves, deteriorating air quality, extreme weather events, and changes in meteorological patterns that alter the transmission of vector-borne diseases, reduce water quality, and compromise food security [30]. Solutions to these problems include the government implementing heat warnings in geographically vulnerable areas and increasing access to prenatal care by building healthcare facilities in these areas. These areas should specifically be targeted for awareness activities and volunteer capacity building toward the health impact of extreme temperatures.

The government, policymakers, and other stakeholders should further try to implement water-containment plans, construct flood control structures, and improve access to healthcare. Taking inspiration from successful policies in other countries, Pakistan can adopt effective strategies to mitigate the impact of CC. Finland has successfully implemented CC thanks to good administration, supportive public officials, and the inclusion of stakeholders [31]. Precautionary measures and public awareness campaigns should be implemented to minimize the adverse effects of heavy rainfall. Pakistan is currently undertaking several actions to combat CC. The “Ten Billion Tree Tsunami Programme, Phase 1” was a four-year project from 2019 to 2023, aiming to revive wildlife resources in Pakistan and combat CC [31]. This program was completed in 2023 with the successful enhancement of forest land and rehabilitation of the current forest area [32]. As of yet, no follow-up initiative to continue this program has been discussed. The Ministry of CC has initiated tree planting in approximately 100 districts across the country to increase forestland, as Pakistan's current forest coverage stands at only 5% [33]. The government program also aims to improve sanitation facilities in Pakistan, as poor sanitation is linked to several diseases, including dengue, cholera, malaria, and diarrheal diseases [32]. Other projects that have been initiated by the Pakistani federal government and provincial departments include the Clean Green Pakistan Index, Ecosystem Restoration, Climate-Resilient Urban Development, and the Green Building Code [34].

Strengths and limitations

This study is the first and largest nationwide quantitative attempt to assess the KAP of the general population of Pakistan on CC. Online and in-person interview data collection methods were used, leading to wider coverage of participants in terms of demographics and region. The questionnaire was administered in both official languages of Pakistan, Urdu and English. Furthermore, the exhaustive questionnaire covered climate hazards, health effects, and other aspects of attitudes and practices.

This study is prone to limitations that were minimized through various processes. The sample size is notably large but might not be adequate to fully represent the entire Pakistani population; therefore, the generalizability of the findings should be considered with caution. Given the situation arising from the floods in 2022, the concern toward CC may be exaggerated because of recency bias. Moreover, this study adopted a self-administered, nonrandomized convenience sampling method. Also, we did not have a preflood KAP study to determine the actual prevalence of common knowledge. Additionally, since it was administered online, there are chances that multiple people might have submitted multiple responses.

## Conclusions

This large cross-sectional study reported an encouraging prevalence of knowledge of CC, positive attitudes, and practices toward CC, with an interest in learning and doing more to address the health effects of CC. CC poses a global threat, particularly alarming in developing countries like Pakistan, where weak infrastructure and limited resources amplify its effects. Given the ongoing global CC trajectory and the forecast of monsoon seasons of similar intensity in Pakistan, identifying demographics with less CC knowledge poses the necessity for targeted educational initiatives to improve public awareness. Policy frameworks should prioritize the dissemination of accurate information through accessible and widely used media platforms while making sure that misinformation is tackled. Additionally, interventions centered around educational institutions could play a pivotal role in fostering CC literacy from an early age.
